# Nécrose utérine suite à une combinaison de compressions utérines et de ligature vasculaire lors d'une hémorragie post-partum: à propos d’un cas

**DOI:** 10.11604/pamj.2020.37.279.26788

**Published:** 2020-11-26

**Authors:** Kamal EL Moussaoui, Najia Zraidi, Aziz Baidada, Aicha Kharbach

**Affiliations:** 1 Département de Gynécologie Obstétrique, Maternité Souissi, CHU Ibn Sina, Rabat, Maroc

**Keywords:** Hémorragie de la délivrance, atonie utérine, capitonnage utérin, nécrose utérine, *case report*, Delivery hemorrhage, uterine atonia, uterin upholstery, uterine necrosis, case report

## Abstract

L'hémorragie de délivrance reste la première cause de mortalité maternelle au Maroc, c´est une urgence obstétricale qui nécessite une prise en charge rapide, efficace et multidisciplinaire. Dans les cas d´hémorragie du post-partum grave rebelle au traitement médical, les progrès de la radiologie interventionnelle et surtout des techniques chirurgicales ont fourni des alternatives sûres et efficaces à l'hystérectomie d´hémostase. On rapporte un cas de nécrose utérine suite à un traitement conservateur chirurgicale de l´hémorragie de délivrance à base d´une combinaison de technique de compression utérine type B-Lynch et triple ligature vasculaire de Tsirulnikov. La patiente a présenté un tableau de sepsis a J4 du post-partum avec un état fébrile et des douleurs pelviennes et un syndrome inflammatoire biologique qui s´est aggravé 48 heures après par l´apparition de contracture abdominale. Le scanner abdomino-pelvien a montré des bulles de gaz dans le myomètre utérin évoquant une nécrose. Une laparotomie exploratoire a été réalisée, l'exploration a révélé une nécrose complète de l'utérus. Les techniques chirurgicales des sutures de compression utérine jouent un rôle majeur dans l'arsenal thérapeutique de l'hémorragie post-partum pendant la césarienne. Elle permet, en complément ou en alternative à la ligature vasculaire, la préservation de la fertilité de la patiente mais elle nécessite également une prudence et une surveillance maximale des complications dont la plus grave est la nécrose utérine.

## Abstract (en)

Delivery hemorrhage is the leading cause of maternal mortality in Morocco. It is an obstetric emergency requiring early, effective and multidisciplinary management in patients with severe postpartum bleeding resistant to medical treatment. Advances in interventional radiology and especially, in surgical techniques have provided safe and effective alternatives to hemostasis hysterectomy . We here report a case of uterine necrosis following conservative surgical treatment of delivery hemorrhage based on B-Lynch uterine compression suture combined with Tsirulnikov triple vascular ligation. The patient had sepsis on day 4 after delivery associated with fever and pelvic pain and biological inflammatory syndrome which worsened 48 hours after the onset of abdominal contracture. Abdomopelvic scanner, showed gas bubbles in the uterine myometer suggestive of necrosis. Exploratory laparotomy was performed, which objectified complete necrosis of the uterus. Uterine compression sutures are surgical techniques which play a major role in the therapeutic armamentarium of postpartum hemorrhage during cesarean section. It allows, in addition to or as an alternative to vascular ligation, the preservation of patient’s fertility, but it also requires maximum caution and monitoring of complications, the most serious of which is uterine necrosis

## Introduction

L´hémorragie de délivrance reste la première cause de mortalité maternelle au Maroc [1]. C´est une urgence obstétricale qui nécessite une prise en charge rapide, efficace et multidisciplinaire. Dans les cas d´hémorragie du post-partum grave rebelle au traitement médical, les progrès de la radiologie interventionnelle et surtout des techniques chirurgicales ont fourni des alternatives sûres et efficaces à l'hystérectomie d´hémostase. Au cours des dix dernières années, les techniques de compression utérine ont été décrites et intégrées dans l'arsenal thérapeutique contre les hémorragies post-partum. Cependant, leur évaluation dans la littérature est encore faible. Nous rapportons un cas rare de nécrose utérine suite à une combinaison de sutures de compression utérine et de ligature vasculaire.

## Patient et observation

Il s´agit d´une patiente âgée de 37 ans, sans antécédent pathologique notable. Quatrième gestation 4^e^ part, les trois anciens accouchements étaient par vois basse. L´actuelle grossesse s´est déroulée sans anomalie. La patiente se présente à la maternité à 39 semaines d´aménorrhée +4 jours en début de travail, l´évolution du travail a été harmonieuse jusqu´à une dilatation du col utérin de 5 cm ou elle a présenté une souffrance fœtale aiguë avec une bradycardie sévère objectivée à l´enregistrement du rythme cardiaque fœtale. Une césarienne a été indiquée en urgence, qui a permis l´extraction d´un nouveau-né de sexe masculin Apgar 10/10^e^, poids de naissance: 3800 grammes. La patiente a présenté en peropératoire une hémorragie de délivrance sur inertie utérine. Le massage utérin complété par la perfusion d'ocytocine et l'admission de 5 comprimés en intrarectal de misoprostol (vu la non disponibilité de sulprostone) n'ont pas corrigé l'atonie utérine, et vu que la technique d´embolisation n´est pas accessible dans notre centre on a eu recours à la triple ligature de Tsirulnikov associé à la compression utérine en utilisant la technique B-Lynch en utilisant un fil résorbable Vicryl 1. Le saignement s´est arrêté et la patiente a bénéficiée d´une transfusion de 2 culots globulaires.

L´évolution en post opératoire était normal jusqu´au 4^e^ jours post-opératoire ou la patiente a présenté une fièvre à (39,6°C) associée à une douleur abdominale et une sensibilité abdominale diffuse, les louchies n´était pas fétides et étaient sans métrorragie. A L´hémogramme GB = 28,000/mm^3^, Hb = 10,1 g/dl et une CRP élevée à 283 mg/L. Une échographie pelvienne a été réalisée montrant une légère hématométrie associée à un myomètre légèrement hétérogène surtout au niveau de la face antérieure de l´utérus. La patiente a été mise sous antibiotique à large spectre visant à traiter une éventuelle endométrite. Quarante-huit heures après, l´évolution a été marquée par l´installation d´un état de choc septique avec une tachycardie à 130 battements par minute, une tension artérielle à 80/40 mmHg avec persistance d´une fièvre à (39,2°C) et cliniquement une contracture abdominale. Un scanner pelvien a été réalisé montrant un épanchement pelvien de moyenne abondance et surtout des bulles de gaz au niveau du myomètre en faveur d´une nécrose utérine. Une laparotomie exploratrice a été indiquée en urgence.

**Figure 1:**
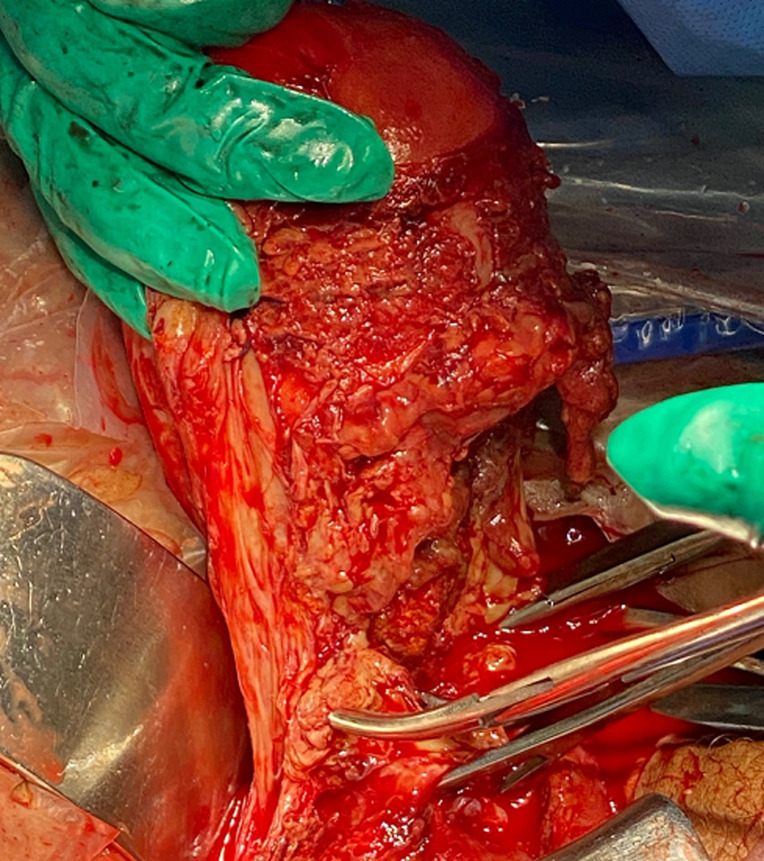
image peropératoire montrant la face antérieure de l´utérus nécrosé et surinfecté

**Figure 2:**
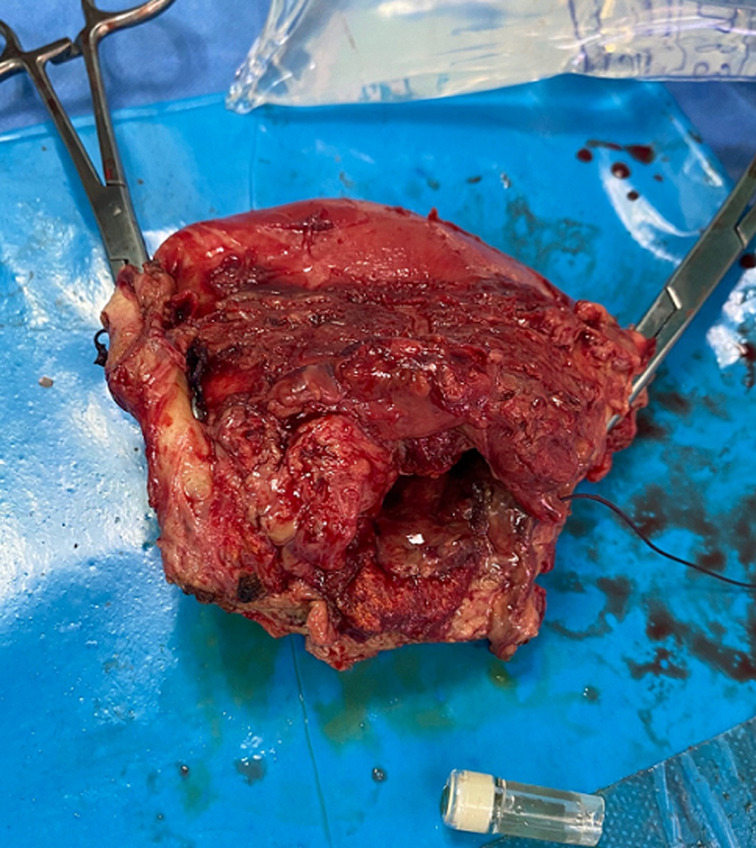
la pièce d´hystérectomie montrant l´importance de la nécrose et de la surinfection de l´utérus

L´exploration chirurgicale trouve une nécrose utérine étendue surtout dans sa face antérieure, une hystérographie ouverte partiellement au centre et surinfectée avec la présence de fausses membranes occupant la totalité de la face antérieure de l´utérus (Figure 1). La nécrose a été définitive malgré l´ablation des fils de sutures de ligatures vasculaire antérieurement réalisée. Une hystérectomie totale a été réalisée (Figure 2) avec un lavage abondant au sérum de la cavité péritonéale avec mise en place de drain pelvien retiré deux jours après. L´évolution post-opératoire a été favorable et l´examen anatomopathologiste a confirmé le diagnostic de nécrose utérine.

## Discussion

L'hémorragie du post-partum est définie comme une perte de sang estimée supérieure à 500 ml dans les 24 h suivant l'accouchement vaginal et plus de 1000 ml après une césarienne [2]. L'hémorragie du post-partum reste une urgence obstétricale qui nécessite une prise en charge rapide. Cette prise en charge se basant sur prise en charge médicale (ocytocine, méthyl-ergométrine, misoprostol), une prise en charge non médicale (massage utérin, compression utérine bimanuelle puis compression de l'aorte abdominale), une réparation de lacérations vaginales ou cervicales et une révision utérine manuelle le cas échéant [3].

En cas d'échec de ces mesures, et/ou si l'état de la patiente est instable, un traitement chirurgical s'impose, Il existe de nombreuses techniques chirurgicales pour la prise en charge de l´hémorragie du post-partum et l'hystérectomie reste la solution de référence dans ce contexte. Cependant, de nouvelles techniques chirurgicales conservatrices qui sont plus faciles à exécuter et sont moins agressives ont émergé et sont devenues plus couramment utilisées [4].

La chirurgie de préservation de l'utérus a été définie comme toute chirurgie d’intervention consistant en une ligature des artères pelviennes ou une application de sutures de compression utérine pour obtenir une hémostase tout en conservant l'utérus, par ex. Ligature bilatérale de l'artère hypogastrique, ligature de l'artère utérine, suture de compression utérine B-Lynch, triple ligature de Tsirulnikov. La triple ligature de Tsirulnikov est une technique chirurgicale simple et conservatrice de l´utérus consistant en une ligature bilatérale des ligaments ronds, utéro-ovariens ligaments et artères utérines [5]. La technique de B-Lynch: pratiquée pour la première fois en 1989 par Christopher B-Lynch chez une femme qui a refusé l'hémostase d'hystérectomie, pendant la césarienne, avec une résistance de suture résorbable 1 ou 2 et aussi longtemps que possible, qui est appliqué autour de l'utérus comme les sangles d'un sac à dos [6]. Suture de Cho (sutures carrées):d’origine coréenne cette technique a été présenté par Cho JH qui consiste à appliquer ensemble les parois antérieures et postérieures de l'utérus par sutures multipoints avec cadre transfixant; point Pereira souvent cité qui combine plusieurs sutures, verticales et une transfixion sous-séreuse horizontale [7].

Bien que ces techniques de compression et de ligature utérine aient été mal évaluées, la facilité de mise en œuvre a permis leur rapide diffusion dans le monde entier. En conséquence, certaines complications sont survenues: pyrométrie, érosion de la sangle à travers la paroi utérine, ischémie utérine, nécrose utérine, synéchie. Néanmoins la fréquence de telles complications reste incertaine compte tenu de l'absence de gros rapports dans la littérature concernant ces procédures, mais pourrait être de 5 à 7% [8].

La nature des fils utilisés (durée de résorption) et le degré de tension initial des points sont deux éléments pouvant expliquer la différence en termes d'ischémie. La technique en elle-même pourrait avoir un effet sur la survenue de la nécrose: une compression uniforme n'interrompant pas la vascularisation pariétale en totalité (notamment en évitant les sutures dans le sens horizontal et en les réalisant seulement dans le sens vertical) pourrait diminuer ce risque [9]. La mise en place correcte des points de compression (seule ou en association avec d'autres procédures hémostatiques) de telle sorte que la reperfusion du myomètre par le réseau anastomotique collatéral est préservée peut diminuer le risque de nécrose [10]. Enfin, la combinaison avec d'autres procédures hémostatiques entraînant une interruption totale du réseau anastomotique de l'utérus, comme ce fut le cas avec notre patiente [10].

Concernant l'association des techniques chirurgicales conservatrices, il reste peu à évaluer. La préférence est à la triple ligature distale type Tsirulnikov complétée si nécessaire par un B-Lynch, modifié selon Hayman (utérus fermé) [11]. L'imagerie joue un rôle important dans le diagnostic. L´échographique est la première ligne car elle montre un grand utérus avec une paroi redessinée et une image hétérogène est associée à la présence d'air dans l'utérus. L'utilisation de la tomodensitométrie est également souvent utile, car dans notre cas, elle a révélé une nécrose utérine avec la présence de bulles de gaz dans le myomètre et dans l'endomètre et un manque de rehaussement du myomètre.

## Conclusion

Les techniques chirurgicales des sutures de compression utérine jouent un rôle majeur dans l'arsenal thérapeutique de l'hémorragie post-partum pendant la césarienne. Elle permet, en complément ou en alternative à la ligature vasculaire, la préservation de la fertilité de la patiente mais elle nécessite également une prudence et une surveillance maximale des complications dont la plus grave est la nécrose utérine. A suspecter devant tout tableau associant les douleurs abdominales, la fièvre et le syndrome inflammatoire après la chirurgie.
